# Structure-Integrated Thin-Film Supercapacitor as a Sensor

**DOI:** 10.3390/s22186932

**Published:** 2022-09-13

**Authors:** Jan Petersen, Alexander Kube, Sebastian Geier, Peter Wierach

**Affiliations:** 1 German Aerospace Center, Institute of Composite Structures and Adaptive Systems, Lilienthalplatz 7, 38108 Braunschweig, Germany; 2 German Aerospace Center, Institute of Engineering Thermodynamics, Pfaffenwaldring 38–40, 70569 Stuttgart, Germany

**Keywords:** power composite, structural supercapacitor, strain sensor, multifunctional material, thin film, carbon electrode, composite structure, energy storage

## Abstract

Today, aircraft composite structures are generally over-dimensioned to avoid catastrophic failure by unseen damages. This leads to a higher system weight and therefore an unwanted increase in greenhouse gas emissions. To reduce this parasitic mass, load monitoring can play an important role in damage detection. Additionally, the weight and volume of future aircraft structures can also be reduced by energy storing and load carrying structures: so-called power composites. In this study a novel method of combining both approaches for maximum weight reduction is shown. This is achieved by using power composites as load monitoring sensors and energy suppliers. Therefore, supercapacitors are integrated into fiber reinforced polymers and are then used to investigate the mechanical load influence. By using four-point bending experiments and in situ electrochemical impedance spectroscopy, a strong relation between the mechanical load and the electrochemical system is found and analyzed using a model. For the first time, it is possible to detect small strain values down to 0.2% with a power composite. This strain is considerably lower than the conventional system load. The developed model and the impedance data indicate the possibility of using the composite as an energy storage as well as a strain sensor.

## 1. Introduction

Today, an increasing number of products are equipped with electric systems, leading to a higher system weight and volume, e.g., assistance systems and consumer electronics in automotive and aviation industries [1,2]. To buffer electrical energy, provide electrical systems in standby, or allow electrical peak power, devices such as batteries and/or (super)capacitors are being used. These energy storage devices normally consist of approximately 30 wt.% of housing material [3], which is parasitic for the overall system. One approach to reduce system mass and volume is the structural integration of electrical energy storage in order to obtain a component that can store electric energy and simultaneously transfer mechanical load [4]. Parts of the load-carrying structure can assume energy storage functions, e.g., act as housing components for energy storage, while the energy storage materials can also bear mechanical loads. Through integrating energy storage devices into structures, huge benefits can be gained, such as decentralizing electronic systems, which allows wiring effort, cable weight, and assembly times to be reduced as, e.g., no compartments for energy storage need to be installed. By now, several integration routes to integrate energy storage in composite structures have been described. Adam et al. introduced a categorization of integration degrees (DIO) [5]. Currently, the two most prominent integrations are the integration of thin layers into the composite structure (DIO II) [3,6] and the integration of energy storage: the composite structure (DIO III) itself [7].

However, integrated energy storage can not only be used to store electric energy, it is also possible to use it as a load monitoring system. In general, the design of mechanical structures (e.g., an aircraft) are driven by damage factors. Structures are often over dimensioned to avoid catastrophic failure by unseen damage with increases in the used amount of material. Condition monitoring offers the possibility to reduce that weight. In case of a fin, a reduction of 5 wt.% was calculated for a system based on piezo ceramics [8]. We investigate the possibility of using structure-integrated energy storage itself as a static load monitoring system. With such a technology, several applications are realizable, for example, monitoring pressurized tanks to investigate their state of health or their filling level. This could also be the fuselage of an airplane or the load of an aircraft’s wing to see its deflection during flight. Information on structural loads like this could then be used to adjust maintenance intervals. In the past, solid state energy storage was characterized under mechanical pressure [9,10]. Lapcinskis et al. [9] described a change in capacitance in relation to mechanical pressure without a deeper look at the mechanisms, while Li et al. investigated a carbon supercapacitor with electrochemical impedance spectroscopy and found a link between mechanical load and the interfacial resistance of the electrode and the electrolyte [10]. In 2018, Dai et al. analyzed a supercapacitor during high g-impact. They built a model to analyze g-forces with the signal from the discharge potential of a supercapacitor [11]. The influence of mechanical deformation was described by Malekian et al. [12], with the outcome that big pores of the electrodes show a greater change in volume compared to smaller ones. All four studies carried out their analyses with conventional energy storage, not with structure integrated ones. In 2021 Roh et al. looked at structure integrated supercapacitors under mechanical load. They analyzed the electrodes resistance of tensile and in three-point bending (3PB) samples [13]. No investigation of the electrochemical behavior under mechanical load was carried out. In this study, thin-film supercapacitors were integrated into glass fiber reinforced polymers (GFRP). Supercapacitors are electrochemical storage devices that can provide and store electric energy in a so-called Helmholtz double layer. Compared to a battery, this is achieved approximately ten times faster [14,15]. The reversible charge separation in the double layers makes the supercapacitors very durable [16], an important aspect for a long-life, maintenance-free multifunctional material [17], which is of high importance since mechanical structures should last a long time.

The supercapacitors in this work were developed to withstand both the conditions of the manufacturing process for composite structures and the subsequent mechanical loads. After producing the multifunctional composite, it was characterized by four-point bending (4PB) tests, according to the DIN EN ISO 14125 test standard. 4PB was used instead of 3PB due to the mechanic effects evolving in the material. On the tensile side of a sample in 3PB, there is tensile, shear forces, bending and even pressure from the load entry. The pressure side behaves similar, except there is pressure instead of tensile. Compared to that, 4PB does not demonstrate pressure through load entry, nor does it have a shear force. This makes it easier to analyze the material and the load-dependent effects on the energy storage. Bending experiments were chosen over other ones, e.g., pure tension or shear, due to their variety. They give information on mechanical tension and the compression condition at the same time. Compared to previous work [13], in this study, the electrochemical behavior of the whole supercapacitor was analyzed, not only the change in the electrode’s resistance. The effects of mechanical load are explained based on several experiments and a mathematical model.

To investigate the influence of the mechanical load on the supercapacitors, an in situ electrochemical characterization was performed. Here, the mechanical tests were paused and kept at a constant force level, while an impedance analysis of the supercapacitors was performed at the same time. This pause is needed, since the impedance spectroscopy takes several minutes to perform. To understand the influence of the mechanical load, an electric equivalent circuit model (ECM) was established based on the physical basis of the used energy storage. The parameters to describe the ECM were determined by fitting them to the measured data. Through additional experiments such as a porosity measurement and EIS of supercapacitors in various states, the ECM was analyzed. With that information, it is possible to further investigate the mechanisms taking place in the supercapacitor as a result of the mechanical load.

## 2. Materials and Methods

### 2.1. Materials

The multifunctional composite is made from GFRP, generally used for load transfer, and comprises two integrated thin-film supercapacitors (ITFC) used for energy storage, as well as sensors. GFRP was chosen over carbon fiber reinforced polymers due to its electric isolation characteristics. The cell components, as well as the composite material, is described in the next sections followed by a chapter detailing the manufacture of the integrated energy storage.

### 2.2. Cell Components

Aluminum sheets coated on both sides with activated carbon (AC) were used as electrodes for the supercapacitor. The AC is located on both sides as the electrodes are delivered in that state (supplied by Skeleton, Tallin, Estonia). The collector thickness and the electrode thickness were 20 µm and 150 µm, respectively. To improve mechanical bonding between the electrode and the GFRP, the AC was removed on one side by grinding, creating a rough surface. The electrodes were separated by a polymer separator (Celgard 3501-4000M-AS40, provided by Gelgard LLC, Charlotte, NC, USA). Both materials were stored in a dry atmosphere (0.3% rH.) at room temperature.

1-Ethyl-3-methylimidazolium bis(trifluoromethylsulfonyl)imide (EMIM TFSI) (supplied by IoLiTec Liquid Technologies GmbH, Heilbronn, Germany) was used as the electrolyte. Before use, the IL was dried using a desiccator.

### 2.3. Structural Components

To provide housing for the supercapacitors and transfer mechanical load, a composite structure with 0/90° fiber orientation was used. The material, Hexply913, is produced by Hexel Corporation, West Valley City, UT, USA. It is a glass fiber reinforced polymer with uni-directional fiber orientation and a layer thickness of 0.125 mm. The material was stored at −25 °C until it was used.

### 2.4. Electrochemical Methods

Electrochemical impedance spectroscopy (EIS) was used to investigate the impedance spectra of the energy storage devices. The EIS (10 mV amplitude, 0.1 Hz–1 MHz unless otherwise stated) was performed during the mechanical experiment to analyze the influence of the mechanical load on the impedance behavior of the supercapacitor. A hybrid EIS was used instead of a potentiostatic or a galvanostatic to prevent the energy storage from drifting or non-linear behavior. A potentiostatic EIS could lead to a drifting cell, as the charge current is higher than the discharge current. This is based on the circumstance that there are, e.g., ohmic losses. By using a galvanostatic EIS, this drifting can be avoided, but there is the possibility that the potential changes rapidly when the cell has a significant change in impedance. This would lead to a non-linear measurement. To avoid these unwanted effects, a hybrid EIS was chosen: a current controlled experiment, with a constant potential amplitude set by the user. All electrochemical measurements were carried out in a four-probe setup.

### 2.5. Composite Design, Assembly, and Preparation

**Figure 1:**
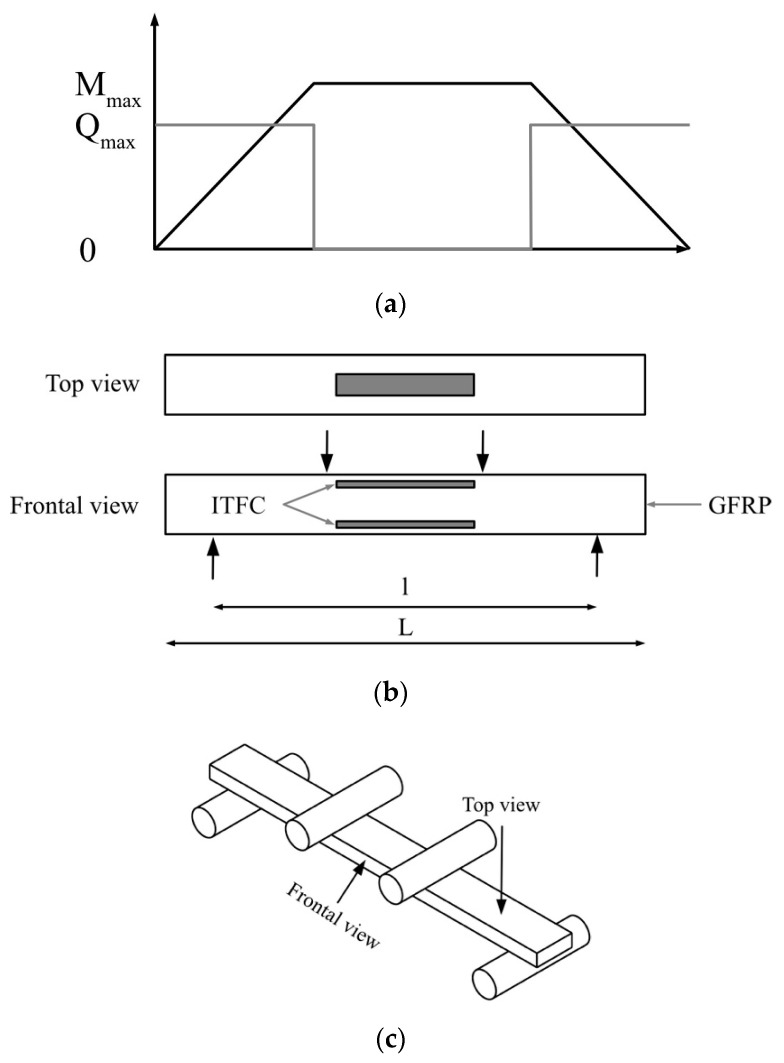
Schematic illustration of the ITFC in the GFRP with lateral force and moments. (**a**) shear forces and moments in the 4PB sample. (**b**) The ITFC is in the area with maximum bending moment and no shear forces. A top view of the sample is shown in the middle, while the frontal view of the sample can be seen on the bottom. The ITFC is gray, the GFRP white with a black border. (**c**) Three-dimensional sketch of the sample in a 4PB setup to show the frontal and top view.

The specimen design was strongly influenced by the German industry standard (DIN ISO 14125 [18]), which gives information on how to perform 4PB tests with GFRP. The length and size of the 4PB sample were adjusted according to Appendix A of the standard. The used width, thickness, and length were 15 mm, 5.3 mm, and 160 mm (Figure 1), respectively. These geometric data were chosen in order to enable the integration of the ITFC with the focus on manufacturing by hand. Smaller sizes would not be manageable. The thickness is defined by DIN ISO 14125 [18] based on the width and length.

Figure 1 schematically shows a GFRP sample with two ITFCs, sideward. The ITFC is located near the outside, in the third layer of the composite. As the current collector is an aluminum sheet with low thickness, it is possible to fold and thread it to the outside of the composite, so as to not to interrupt the fibers. This is important for an even stress distribution and for the electric contact of the instrument to the supercapacitor.

The four arrows in Figure 1 show the position and direction of the force through the 4PB test. They represent the point in the experiment where the force is initiated. A constant bending moment (Figure 1, M) in the area of interest with no shear force (Figure 1, Q) is typical for 4PB.

As shown above, one ITFC is integrated on the tension and one on the compression side of the sample. To determine the strain in the ITFC, the mathematical equation ($\epsilon \left(y\right)=\epsilon_{0}\cdot z/z$) was used based on the strain deformation on the outside of the composite, which was measured by strain gauges. A total of four strain gauges were placed on one sample, two on the compression and two on the tension side, to obtain information on the evenness of strain distribution during the experiment and the strain itself. The surface area where the strain gauges are located was grinded by hand. The strain gauges were then attached with glue to the area where the ITFC is located. Based on the composite layup, the position in the z direction and the strain in the outer layer of the experiment, the strain in the supercapacitor can be calculated to be 0.33–0.36%, for an outer strain of 0.4%, and 0.17–0.18%, for an outer strain of 0.2%.

As the composite is symmetric, the amount of the strain is the same for compression and tension, but with different signs.

The GFRP preparation is a typical prepreg layup made by hand. The supercapacitors are located in two layers with a window in it. Before preparation, the supercapacitor was placed in a vacuum for 24 h with an electrolyte on the AC side. This step is necessary for the electrolyte to infiltrate the electrode. Afterwards, two electrodes, separated by a Celgard separator, were placed in the composite. A panel made of twelve supercapacitors, six on the compression and six on the tension side, was assembled.

The prepreg was then cured according to the technical datasheet in a two-step autoclave process at 6 bar pressure and 125 °C maximum temperature. After curing, the composite plates were cut into the specimen geometries, according to the standards for 4PB tests.

### 2.6. Equivalent Circuit of the Integrated Supercapacitor

**Figure 2:**
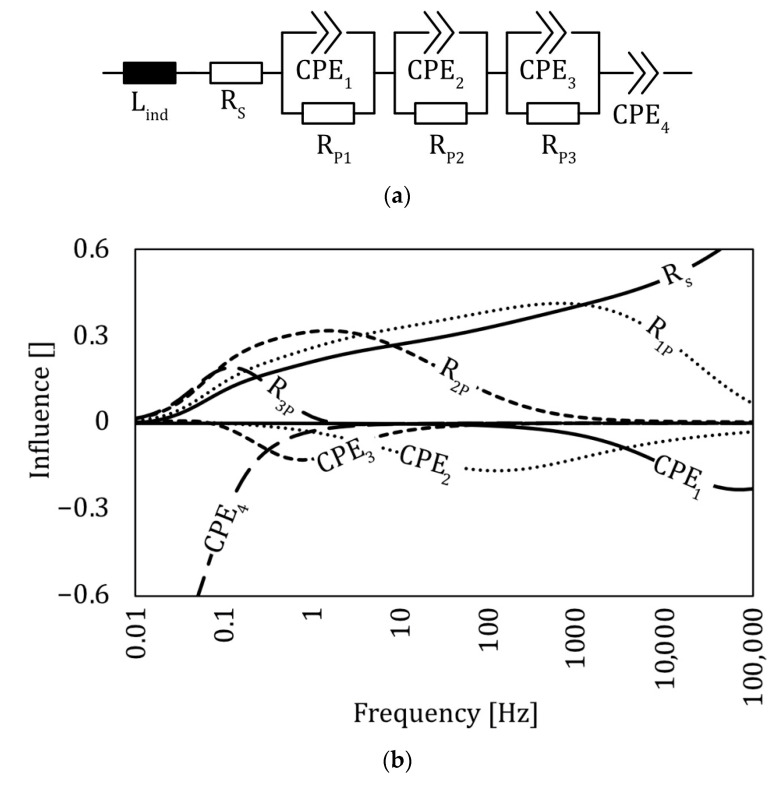
Equivalent circuit model with corresponding influence plot (**a**) Equivalent circuit used to fit the measured data to elements describing the supercapacitor. (**b**) Influence of the elements used on the equivalent circuit in relation to the excitation frequency. Each element has a frequency depended influence on the whole impedance of the supercapacitor. e.g., CPE1 leads to a greater change in impedance at high frequencies compared to CPE4. Negative influence means it is reducing the impedance, positive influence increases the impedance. Influence is a dimensionless value.

A schematic representation of the equivalent circuit model to a supercapacitor is visualized in Figure 2a. It was used to analyze the effects of load and temperature on the integrated supercapacitors. The model is based on the physical background of the produced supercapacitor, which is described in the following paragraph and analyzed more deeply in the Section 4.

All data investigated in this work are analyzed by fitting to the equivalent circuit (Figure 2a). A series of three RQ elements, each consisting of a CPE and a resistor (R) in parallel, was used to describe the electrodes pore network, as the electrode is based on activated carbon. In the literature, a CPE describes non-ideal behavior resulting from, e.g., the surface roughness, distribution of reaction rates, varying thickness or composition, or non-uniform current distribution [19,20,21,22,23,24,25]. An AC electrode has a specific surface of approximately 1000–3000 m^2^/g [26], consisting of a pore network made of carbon particles. This structure has a non-uniform surface, which can therefore be modeled using a CPE element. R_P,n_ describes the corresponding charge transfer reactions, while R_s_ is used to represent the sum of all ohmic resistances present in the supercapacitor such as, e.g., the electrode, the electrolyte, and the current collector. The double layer capacity is parameterized by a single CPE element (CPE_4_). Since the electrolyte is an ionic liquid and the polarization is negligible (cell potential of 0 V), the double layer can be treated as a single layer [27,28]. L_ind_ is used to describe the inductive behavior resulting from cables and the connection of the probe, as well as the current collector itself.

Each element in the circuit can be described mathematically by (Equations (1)–(5)) [29]:

(1) $$Z_{S}=R_{s}$$

(2) $$Z_{CPE-R}=\frac{Z_{CPE}Z_{P,n}}{Z_{CPE}+Z_{P}}$$

(3) $$Z_{CPE,g}=\frac{1}{{\left(j\omega \right)}^{\alpha }Y_{g}}$$

(4) $$Z_{P,n}=R_{P,n}$$

(5) $$Z_{IND}=j\omega L$$

$R_{S}$ being the serial resistance, $R_{P,n}$ the parallel resistance for charge transfer, $Y_{0}$ and α the constants of the CPE, L the inductance, and ω=2πf with f being the frequency.

The description of the equivalent impedance $Z_{ec}$ is expressed by:

(6) $$Z_{ec}=Z_{IND}+Z_{S}+\overset{3}{\underset{n=1}{\sum }}Z_{\left(CPE-R\right)n}+Z_{CPE,g}$$

Therefore, the electrochemical equivalent circuit can be expressed by:

(7) $$Z_{ec}=j\omega L+R_{s}+\frac{1}{{\left(j\omega \right)}^{\alpha }Y_{g}}+\overset{3}{\underset{n=1}{\sum }}\frac{\frac{1}{{\left(j\omega \right)}^{\alpha }Y_{0}}R_{P}}{\frac{1}{{\left(j\omega \right)}^{\alpha }Y_{0}}+R_{P}}$$

### 2.7. Electrochemical Tests

The electrochemical tests were performed with an electrochemical workstation (REF3000, Gamry, Warminster, PA, USA). For each of the twelve capacitors, an impedance measurement was carried out before and during the mechanical testing. EIS was conducted at 10 mV amplitude in the frequency range of 100 mHz up to 1 MHz for the in situ four-point bending tests and 10 mHz–1 MHz for the in situ compression test. The upper limit was chosen because the EIS data shows inductive behavior above 1 MHz, while the lower limit was chosen due to time constrains. Additionally, a low frequency impedance was carried out in the frequency range of 1 mHz–1 MHz to analyze whether the supercapacitor reveals diffusion. All tests were conducted within the accuracy plot of the manufacturer, giving an error smaller than 1% [30]. Since the measurements performed as part of the electrochemical tests take a certain amount of time and are sensitive to changes, the force levels were kept constant during the mechanical tests.

To reach a quantitative statement and to analyze the electrochemical results in relation to the mechanical load, an equivalent circuit (Figure 2) was modeled using software (RelaxIS3, RHD Instruments GmbH & Co. KG, Darmstadt, Germany). The result of the model was fitted to meet the measured impedance taken from the samples.

### 2.8. Four-Point Bending Tests with In Situ EIS

The 4PB tests were performed with a standard testing machine (Zwick Z005, Zwick GmbH & Co. KG, Ulm, Germany). Force was measured by a 5 kN load cell (BZ2-MM14760.ZW02, Zwick GmbH & Co. KG, Germany), while strain was measured with strain gauges (DMS, XY-F/DMS, Höttinger Baldwin Messtechnik GmbH, Hemmingen, Germany). The sample deflection is recorded by an inductive linear position sensor (WA 50, type K-WA-U-050-W-32K-K4-F1-2-8-10, Höttinger Baldwin Messtechnik GmbH, Germany).

**Figure 3:**
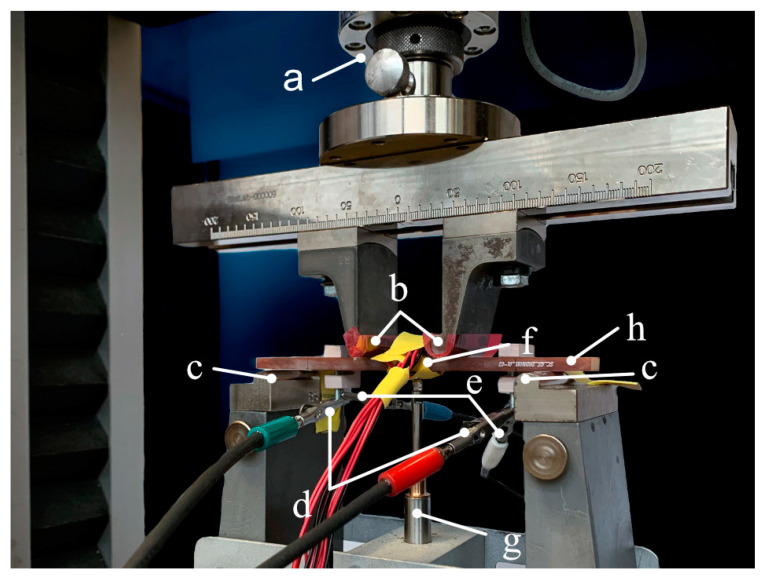
Four-point bending test setup. The GFRP beam (h) with ITFC is shown between the four force entry points (b,c). Four cables (d,e) are connected to the sample for EIS measurement. The power (d) and sense (e) cables are placed separately so as to not interfere with each other. Deformation is detected with a linear position sensor (g), touching the bottom of the sample. Strain is detected with four strain gauges (f) whose cables can be seen running from the middle of the sample to the bottom of the image. Mechanical force is measured via a load cell (a).

The 4PB test setup is shown in Figure 3. The GFRP beam (h) is positioned on top of two bearings (c). Additionally, two bearings (b) transfer the force into the sample. Force is measured with a load cell (a), shown on the top of the image. The cables for EIS are connected to the sample via clamps (d, e). Power (d) and sense (e) cables are separated so as not to interfere with each other. Strain is detected with four strain gauges (f), two on the bottom, and two on the top of the GFRP beam. The strain sensor cables run downward from the sample. A four-point connection is used to reduce contact and cable resistance. Deflection is detected with a linear position sensor (g).

In order to analyze the change in impedance with regard to strain and, therefore, mechanical load, three load steps are used. A linear change in load whilst simultaneously measuring the impedance was not performed, as the EIS measurements take approximately 5 min. The last load step was set to reach a strain of 0.4% in the outer layer of the sample. This resulted in a strain of approx. 0.35% in the supercapacitor. The first step was 10 N, leading to nearly no strain in the sample. This load step was used to have a defined starting point. The second step was set to 0.2% outer strain or 0.18% in the ITFC. Load steps are chosen to show a realistic scenario in the elastic range of the composite. Some load steps up to 0.4% strain were performed before the actual experiment to check whether the system behaves elastic.

To reduce measurement errors, the following steps were carried out. At first, EIS was recorded multiple times to make sure that there is no change in impedance for the same load scenarios. This approach should ensure that the cell and the mechanical system are in a steady state. Additionally, the same load steps were recorded multiple times to prove the reproducibility of the impedance plots of the same load step. This was carried out to check the linear elasticity of the mechanical system. In a second step, the sample was analyzed once in and once outside of the test machine to determine whether the machine itself influences the impedance spectra. The cables of the potentiostat were placed according to the manufacture’s recommendation to reduce stray inductance [31]. The third and last step was a regular calibration of the potentiostat and the whole test machine with its force and position sensors.

The experiment took place in a defined and stable test environment at 23 °C and 50% rh.

### 2.9. Preliminary Pressure Tests Analyzed by In Situ EIS

To examine the influence of a force perpendicular to the electrode, a mechanical experiment was set up (Figure S1). For this purpose, an aluminum container was filled with the electrolyte EMIM TFSI. Two supercapacitor electrodes were added to the tray. They were separated by the above-mentioned separator. To prevent contact between the container and the electrodes, two PTFE plates were used as a force transmission element. They were placed beneath and on top of the supercapacitor. The pressure was generated by a test machine (Zwick Z005, Zwick GmbH & Co. KG, Germany) and measured by a mechanical load cell (XforceP, 500 N, Zwick GmbH & Co. KG, Germany).

The EIS was performed with a potentiostat (REF3000, Gamry, Warminster, PA, USA) connected to a four-probe setup. As mentioned before, power and sense cables were separated to prevent inductive errors. Force was held constant ten times in the range of 0.01 mPa up to 1 MPa, with a step width of 0.1 MPa. During each pause, in which the force was held constant, the impedance spectroscopy occurred. The frequency range was 10 mHz–100 kHz, with an amplitude of 10 mV. The experiment was carried out at room temperature (23 °C) and 50% rh. The upper frequency of 100 kHz was chosen because long cables (1.5 m) are used in this setup, leading to a reduced maximum frequency.

### 2.10. Optical Displacement and Deflection Detection

**Figure 4:**
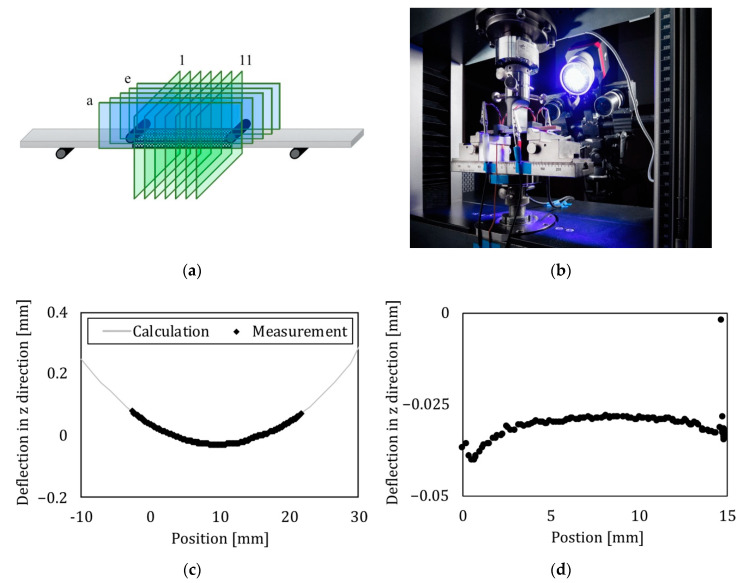
Optical analysis of a bending beam to investigate the geometric deformation. (**a**) Position of the sections where the deflection is extracted. Blue shows the sections in beam direction, green the sections between the inner load entry points. (**b**) Optical system to measure the displacement of the samples during mechanical tests. The system uses two cameras to get the displacement information. For better contrast, the sample has a random pattern on the surface and the surface is illuminated with blue light. (**c**,**d**) Deflection of the beam in the inner section between the force entry points. The curve is one of six similar deflection lines. C shows the bending of the beam, while D is the buckling of the surface.

To obtain a deeper insight into the deformation of the bending beam, an optical system (ARAMIS, GOM GmbH, Braunschweig, Germany) was used to analyze the displacement, as well as the deflection. The focus of interest is the change in geometry of the surface of the beam in the area (Figure 1) of the supercapacitor. This area is located between the two inner force entry points (Figure 3). By applying a random pattern of black and white dots to the surface of the bending specimen, the optical system can track each single point with a system of two cameras (Figure 4b). Before use, the system was set up and calibrated to deliver a measurement with an accuracy of approximately 3 µm in deflection. To reach such a high precision, only a small area can be analyzed. The area between the two upper bearings was observed in accordance with these requirements. The aim of this measurement was to find possible buckling of the surface where the ITFC is located.

### 2.11. Determination of the Electrode Pore Size Distribution

The pore size distribution was determined using mercury porosimetry, which can present information on the pore volume, pore size distribution, and porosity. During the measurement, non-wetting mercury was forced into the porous system by applying and varying external pressure. By using the Washburn equation, the corresponding pore radius (*r*) can be calculated by:

(8) $$p=\frac{-2\gamma \cdot \cos\left(\Theta \right)}{r}$$

if the applied pressure (*p*), the mercury surface tension (*γ*), and the contact angle (*θ*) are known.

Measurements to determine the pore size distribution were carried out using a Pascal 140 measuring system in combination with Pascal 240 from the company Porotec, Germany.

### 2.12. Statistical Analysis

Here, E is the identifier for an element which can be a resistor or a CPE. m is the index for the element and n the number of the sample. The change in element is calculated with Equation (9), while the mean of all corresponding elements is calculated with Equation (10). N is the count of all samples, in this case 6.

(9) $$E_{m,n,change}=\frac{E_{m}}{E_{m,\;init}}$$

(10) $$E_{m,\;mean}=\frac{1}{N}\overset{N}{\underset{n=1}{\sum }}E_{m,n,change}$$

The error bars indicate the standard error of the mean and are calculated by Equation (11) with the help of Equation (12).

(11) $$s_{m,error}=\frac{s}{\sqrt{N}}$$

(12) $$s_{m}=\sqrt{\frac{\sum^{}{\left(E_{m,n,change}-E_{m,mean}\right)}^{2}\;}{N-1}}$$

## 3. Results

To analyze the effects of mechanical load on a structure-integrated supercapacitor, different experiments are necessary, which are divided into the characterization of the electrode material, an equivalent model creation with a subsequent analysis, and mechanical experiments to investigate the change in electrochemistry through the change in mechanical load. Three mechanical load scenarios were investigated: (i) an orthogonal pressure setup of the electrodes, (ii) a four-point bending experiment of an integrated supercapacitor on the tensile and (iii) on the compression side.

### 3.1. Characterization of the Electrode

**Figure 5:**
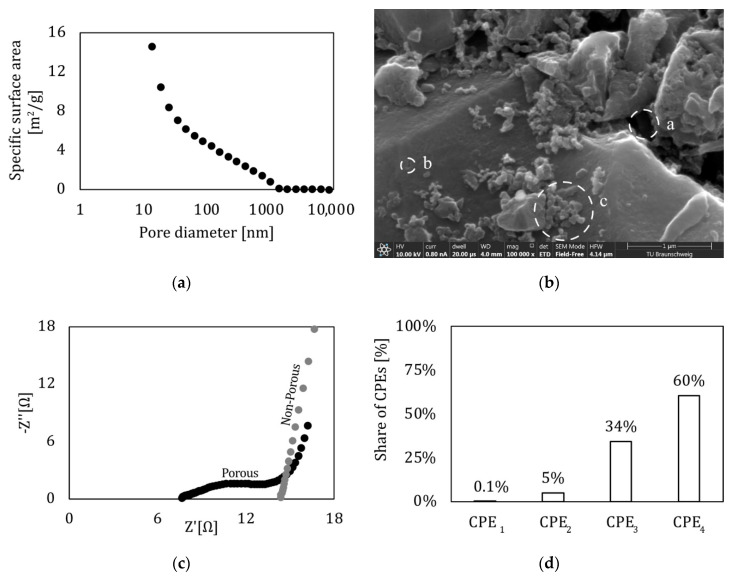
Analysis of the electrode material (**a**) Specific surface area of the electrode material by mercury porosimetry. (**b**) SEM image of the used electrode with different pore geometry and size. Three kinds of pores are identified. (a) big-sized secondary pores (b) small-sized primary pores (c) small-sized secondary pores. (**c**) Impedance spectra of two samples, one with an activated carbon electrode and one with a flat electrode made of aluminum. The porous network of the electrode is represented by the black semi circles (dashed ellipse), which are not present at the flat aluminum electrode. (**d**) Share of CPEs in the equivalent model.

To analyze the surface structure of the electrode, three different techniques were used. The pore size distribution was investigated with the mercury porosimetry method, while the structure was analyzed with a scanning electron microscope (SEM). The last technique was electrochemical impedance spectroscopy (EIS) to obtain information on the electrochemical impedance of the sample. The results of the mercury porosimetry can be seen in Figure 5a. The electrode shows no specific surface area with pores that have a diameter above 1 µm. The share of the pore specific surface area starts increasing linearly at 1000 nm up to 20 nm, where it begins to increase rapidly. The highest specific surface is reached, with pores having a diameter of 10 nm and smaller. It must be mentioned that it is not possible to investigate pores with diameters smaller than 10 nm due to the machine’s restrictions.

To obtain an optical insight into the electrode itself, a SEM image (Figure 5b) of the electrode profile was carried out. Before creating the SEM image, the sample was cooled in liquid nitrogen and broken afterwards. The picture reveals different kinds of pore sizes and micro cavities. In this work, pores or micro cavities consisting of a single element are named primary pores, while the porous system built from agglomerates is called secondary pores. The overall impression of the SEM image is that it is heterogeneous in size and form. Small-sized primary pores (b) can be found as well as small- to medium-sized secondary pores (c). Finally, there are large sized secondary pores (e.g., dashed circle a). Due to the sample preparation, smaller pores could not be visualized in the obtained SEM pictures.

For a deeper understanding, an equivalent circuit was established, consisting of resistors and constant phase elements (CPE). CPEs are used to describe the pore network of the electrode, as well as the capacity of the supercapacitor. Resistors are used for charge transfer and resistance losses. A detailed and more in-depth description of this model is available in “Equivalent Circuit of the Integrated Supercapacitor”. The properties of the CPEs were investigated and compared to the results of the mercury porosimetry analysis (Figure 5a), as well as a frequency analysis (Figure 2b). The link between the ECM, the frequency analysis, and the mercury porosimetry measurement is discussed later in this study. Figure 5d visualizes the share of all CPE elements used to describe the supercapacitor. Each CPE (1–4) is a mean of the six samples taken from the orthogonal pressure test (“Preliminary Pressure Tests Analyzed by in situ EIS”) in a load free state. After calculating the mean of each element, the share (e.g., for CPE_1,share_ = CPE_1_/CPE_total_ with CPE_total_ = ∑CPE_n_, n = 1.4) was calculated. CPE_1_ and CPE_2_ have small values compared to CPE_3_ or even CPE_4_, which has the greatest share.

### 3.2. Electrochemical Model Analysis and Verification

An ECM (Figure 2a) was used to analyze the system in various conditions to see its wide range of applications, which is an indication that the ECM is valid for the used system. Additionally, by varying the operation conditions and the sample properties, the influence of the EIS data in relation to the equivalent circuit elements can be investigated. 

All tests in this chapter are performed in a test cell (TSC Battery, RHD Instruments GmbH & Co. KG, Darmstadt, Germany) at 20 °C (if not noted otherwise), with a potentiostat (REF3000, Gamry, Warminster, USA). The following parameters varied: temperature, polarization of the electrodes, electrode distance, electrode thickness, separator thickness, and electrode material. To study the pore network of the activated carbon (AC), two types of electrodes, one with a pore network and another one without one, were investigated (Figure 5c). One is achieved with a supercapacitor cell made of an activated carbon electrode coated on an aluminum sheet: the current collector, while the other one is a pure aluminum sheet. With the help of this measurement, the three CPE-R elements should represent the interaction between the electrode and the electrolyte, because the area associated with the CPE-R elements (dashed ellipse in Figure 5c) is missing for the electrode consisting solely of the current collector.

EIS measurements at different polarization in the range of 0 V–2 V showed that the supercapacitor has almost no pseudocapacitive behavior. This is based on the assumption that a linear increase in polarization leads to a linear increase in capacity.

The change in the separator thickness demonstrates that the serial resistance (R_s_) is influenced by it. There was a shift in impedance on the real axis, indicating an increase in resistance of 61% when doubling the electrode distance, and an increase of 131% by tripling it.

For the reason of compactness, the results of temperature, polarization of the electrodes, and electrode thickness were not included. The change in temperature is investigated elsewhere [32].

### 3.3. Frequency Analysis of the Equivalent Model

**Figure 6:**
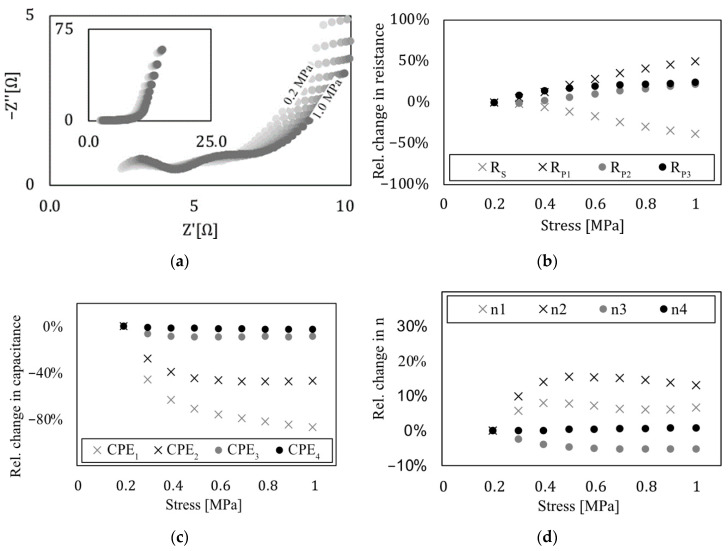
Electrochemical impedance in relation to perpendicular mechanical pressure (**a**) Results from the EIS due to mechanical pressure. (**b**) CPE_n_ obtained from fitting. (**c**) Resistance obtained from fitting. (**d**) Exponent of the CPE obtained from fitting.

A frequency analysis (Figure 2b) of the equivalent model was conducted to further understand the ECM. Therefore, an influence plot was created based on the ECM itself and the fitted data from the pressure test (Figure 6) in the load free scenario. The influence is a dimensionless value describing the impact of each element in the frequency range. It is in the range of −1 to 1. The calculation of the influence is described in the following. By building the partial derivation of the modulus of the ECM’s impedance for each component, the influence can be calculated (Equation (13)). This was carried out for every parameter (all R_Pn_, and CPE_n_) with the fitting parameters of the pure pressure test. An example for the influence parameter of the resistor R_S_ can be calculated according to Equation (13). I_RS_ is the influence factor, *R_S_* the serial resistance and *Z_ec_* the impedance of the equivalent circuit. To create Figure 2b this was carried out for all elements.

(13) $$I_{\mathrm{RS}}=\frac{R_{S}}{\left|Z_{ec}\right|}\cdot \frac{\partial \left|Z_{ec}\right|}{\partial R_{S}}$$

The influence of each element in relation to its frequency is shown in Figure 2b. The influence of all resistors is between 0 and 1, while CPEs are in a range of 0 ... –1. A resistor and a CPE element in parallel are coupled. Each couple influences the impedance in a specific range: couple one in the high frequency range, two in the middle, and three in the low frequency range. The very low frequency is dominated only by CPE_4_. The serial resistance R_s_ has an influence on the highest frequency area. The inductor was not included because it is used to describe wiring effects, which are not influenced by the mechanical load. With that information and the BET measurements, as well as the information from the porous/non-porous investigation, a link between the pore diameter and the elements in the equivalent circuit is found.

### 3.4. Influence of Mechanical Pressure on the Impedance Spectra

The results from the EIS measurement in a setup where the electrodes are in a state of pure compression are visualized in Figure 6a.

The first measurement was carried out at a preload of 0.2 MPa. The pressure then increased up to 1.00 MPa in 0.1 MPa increments. The ECM was fitted to the electrochemical impedance data of every load step. In this chapter, the elements are analyzed with respect to their relative change, meaning relative to their initial state.

A Nyquist plot in Figure 6a visualizes the effect of perpendicular pressure on a supercapacitor. The area of very low frequency (f = 0.01 Hz–0.1 Hz) is visualized on the top left in a reduced plot, as the change due to mechanical pressure is low compared to the mid- to high-frequency areas, described by elements CPE_1_–CPE_3_.

The change in mechanical pressure on those elements is more pronounced compared to CPE_4_’s influence in low frequency areas. Figure 6, c shows the changes in the CPEs for varying pressures. An increase in those elements up to 87% (CPE_1_), 47% (CPE_2_), and 9% (CPE_3_) can be observed. The same can be seen in Figure 6, a by the increasing waviness of the curves. The first two maxima correspond to CPE_1_ and CPE_2_. CPE_3_ influences the rising range in the low frequency range, while CPE_4_ can be seen on the reduced figure as mentioned before. Next is the change in the exponent of the CPE itself. In the case of n_4_, the mechanical load has no significant influence on the parameter; n_1_, n_2_, and n_3_, however, are influenced greatly by the load. The change in CPE, as well as the change in its exponent, both have in common that the characteristic of load influence is not linear. The graph seems to follow a function such as 1/x+a. Pressure influences not only the change in the CPE, but also the resistances. R_s_ becomes smaller with an increasing load, while R_2_–R_4_ behave in reverse (Figure 6b). This results in a shift in the curve’s maxima. The change in all resistances roughly follows a linear function.

### 3.5. Influence of Mechanical Strain in Four-Point Bending Experiment

**Figure 7:**
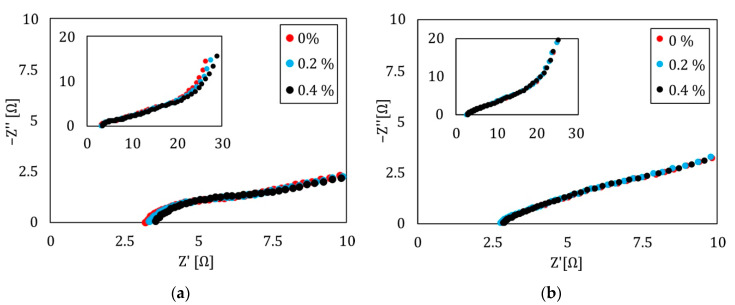
Influence of mechanical force on a structurally integrated energy storage (**a**) EIS plot for three different load situations. Red indicates a load free situation, while blue and black show the EIS results under mechanical load with 0.2% and 0.4% strain. All measurements are performed in a 4PB experiment on the tension side of the sample. Frequency range was 100 mHz–1 MHz. (**b**) EIS plot for three different load situations. Red indicates a load free situation, while blue and black show the EIS results under mechanical load with 0.2% and 0.4% strain. All measurements are performed in a 4PB experiment on the compression side of the sample. Impedance is visualized by rectangles, the phase by circles.

As described in Section 2.8, a four-point bending experiment with integrated supercapacitors was set up and investigated with EIS in different load scenarios. The result of one supercapacitor on the tension side (Figure 7a) and one on the compression side (Figure 7b) is shown here, as an example. The three load steps are visualized by colors, with red being the initial load free state, blue the state with a strain of 0.2% on the surface of the sample, and black the state with an outer fiber strain of 0.4%. Both plots show the high-frequency region, while the entire measured spectrum is plotted smaller in the corner of each diagram.

Naturally, there is a correlation between the mechanical load and the impedance spectra of the supercapacitor on the tension side, while the compression side seems nearly free of any mechanical influence (Figure 7b). On the tension side, there is a change in the whole spectrum which is investigated more deeply with the above-described ECM. To obtain a more general statement, the tension side of all six samples is investigated with the help of EIS and then analyzed by fitting the data to the ECM. Afterwards, the change in each element (E) in relation to its initial state (init) (Equation (9)) is calculated and, on that basis, the mean (Equation (9)) is determined.

**Figure 8:**
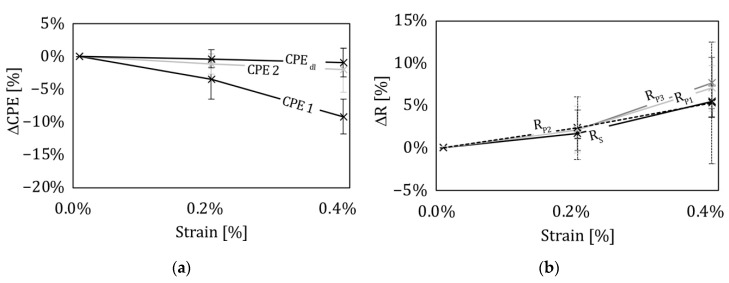
Fitting results of the electrochemical equivalent circuit due to mechanical load in a four-point bending experiment. The figures show the mean of each element based on six samples. Error bars indicate the standard error. (**a**) Change of the constant phase elements through mechanical load. (**b**) Change of the charge transfer through mechanical load.

With the preceding steps, Figure 8 is created, showing the parameters of all elements of the equivalent circuit in relation to the mechanical strain. The change in CPE_3_ is not included because it remains nearly constant. Since an impedance scan is more time-consuming the deeper the investigated frequency is, a low frequency limit of 0.1 Hz was chosen to avoid the settlement effects of the specimen. This reduced the time needed for one EIS from approximately one day (lower frequency 1 mHz) to several minutes. The downside is that information from the low frequency range was lost. To gain insights into low frequency effects, pressure tests in electrolyte baths (Figure 6) were used. To analyze the low frequency domain where CPE_4_ has the strongest influence, a low frequency measurement was performed after the mechanical test with a frequency range of 1 mHz up to 1 MHz, as part of the four-point bending (4PB) measurements. For the reason of compactness, the results of the exponent of CPE_1_ to CPE_4_ are not included, as they show no significant changes with varied mechanical loads. When applying a mechanical load in a four-point bending experiment on the compression side of the sample, it can be observed that CPE_1_ und CPE_2_ decrease with increasing load, or outer fiber strain. The resistances R_S_, R_P1_, R_P2_, and R_P3_ increase with increasing mechanical load.

None of the observed changes are of a linear type. The greatest influence was found at CPE_1_ and R_P3_. It has to be mentioned that elements describing charge transfer now showed deviation from the mean. This should be based on the specimen preparation. This involves manufacturing by hand and therefore each sample is an individual one. To be able to evaluate the mechanical influence on the impedance of an integrated supercapacitor, we built twelve integrated supercapacitors. This reduces the risk of analyzing an outlier.

### 3.6. Optical Deformation Analysis under Mechanical Load

An analysis of the deflection with an optical system was conducted for all six samples. To see only the deformation of the beam itself, the global movement (rigid body motion) of the sample in the observed area is subtracted from the results. 

The deflection of the beam was extracted for different sections of the sample, as shown in Figure 4a. There are six sections in the beam direction (blue) and eleven sections between the inner force entry points (green).

A representative deflection of the beam in the z direction (in force direction) is shown in Figure 4c,d. The overall deflection in the inner area is approximately 0.1 mm (Figure 4c). Furthermore, the sample with the integrated thin-film supercapacitor (ITFC) behaves like a conventional bending specimen. An analytical model based on a bending beam in a four-point bending setup is used to verify the experimental data and is plotted in gray. The measurement overlaps the calculation. Additionally, there is no buckling over the entire sample. As previously mentioned for the bending in the length direction (Figure 4a, lower case alphabetical), the sample changes its shape from a plane surface to a curved one (Figure 4d), typical for a bended specimen. The data visualized is layer c (Figure 4a).

## 4. Discussion

**Figure 9:**
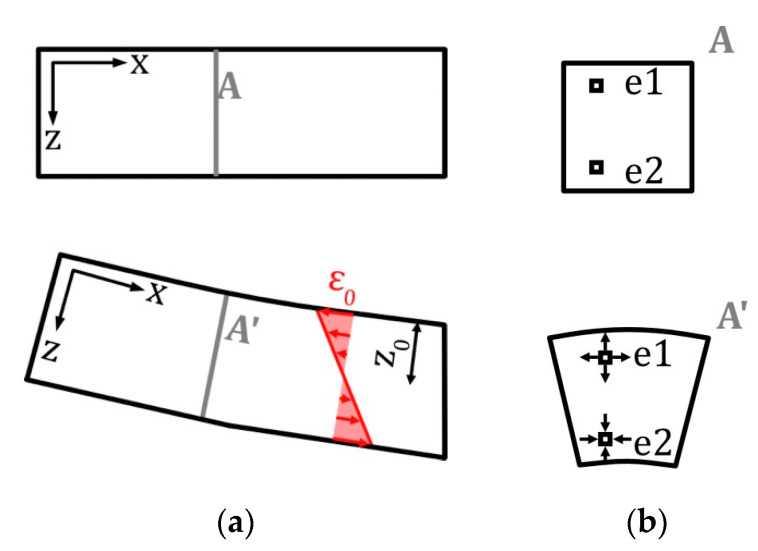
Analytical analysis of a composite bending beam. Without load shown on the top and with load on the bottom (**a**). Stress distribution caused by the constant moment is visualized in red. (**b**) Sections of the bending beam showing two elements (e1 and e2) and their deformation.

The results obviously show the strong reaction of the ITFC placed on the tension side of a four-point bending sample, thus making it usable as a sensor system. On the compression side, only a minor response to mechanical load was observed. For this reason, the discussion focuses on the tension side. The capacity related to CPE_1_ had a great influence on the mechanical load (Figure 8). To analyze the role of CPE_1,_ a deeper look into the distribution of the pore diameter is necessary. The mercury porosimetry measurement of the electrode showed that the material has different pore diameters which are unequally distributed (Figure 5a). Most of the specific surface relies on pores with very small diameters, while big pores represent only a small share of the total porosity (Figure 5a). The same can be seen for the share of the capacitive behavior described by the CPEs. There is an unequal distribution of all CPE elements, with the greatest amount of total capacity linked to CPE_4_. This element influences the impedance spectra in the very low frequency range, while CPE_1_ has its greatest influence in the high frequency domain. (Figure 2) This behavior is a strong indication that CPE_1_ correlates to big diameter pores, as they are able to react to fast current changes [33]. Additionally, the distribution of pore diameters (taken from the porosity measurement) matches the distribution of CPE elements, with the result that CPE_1_ should correlate to big diameter pores. All this information points to the conclusion that the mechanical load coming from a 4PB changes the pores’ geometry. Based on the nature of the bending experiment, the supercapacitor on the tension side is stretched in a lengthwise direction (Figure 9, X-direction), while being compressed orthogonally to it. Figure 9b shows the profile of a bending sample in a load free and bended scenario, with the forces on a small element on the tension (e1) and compression (e2) side. The profile of the supercapacitor on the tension side is deformed by the mechanical load similarly to element e_2_, meaning its profile is under compression load.

The hypothesis is based on the mechanical interaction of the GFRP structure with the ITFC. On the tension side, the energy storage will mostly see tension in a lengthwise direction and compression of its profile. This leads to a compression of the electrode material and therefore the pores, which in turn leads to the described sensory effect.

The compression side shows a much smaller change in impedance due to mechanical load. In that case, the electrode of the sample is compressed by the surrounding GFRP structure in a lengthwise direction. Opposite to the tension side, the profile is in a state where it is dragged to the outside by the GFRP. The further away the electrode material is from the GFRP (electrode material from the inner section of the supercapacitor), the lower the deformation might be, as the electrode consists of agglomerates with only a small possibility to transfer that kind of mechanical load.

Like CPE_1_, CPE_2_ also reacts to mechanical load, but with less intensity. One explanation is based on the pore size. As mentioned before, CPE_2_ corresponds to smaller pores than CPE_1_ and therefore experiences less deformation from mechanical load. This leads to a smaller electrochemical response. CPE_3_ describes the smallest pores in the ECM, which are nearly unaffected by the applied mechanical load. When comparing the results from the pure pressure setup with the results from the bending experiment, we can see that in both cases a mechanical load change leads to a decrease in all CPE elements. The CPEs are affected the most when they are describing big pores.

Besides the change in capacity, a change in resistance was also determined. The resistor R_S_ rises with an increasing mechanical load. This behavior is opposite to what was seen in the pressure tests. Different explanations for this behavior can be found and a more in-depth analysis is required. One reason could be the change in the MacMullin number, a vale describing the ion conductivity of a separator. Throughout the increasing compression of the separator, its MacMullin number could be increased, leading to an increase in the serial resistance R_s_. A reason why this is not present in the pure pressure setup might be that the increase in pressure leads to a reduction in the distance of the electrodes and, therefore, an improvement in R_s_, which overlaps the change in the MacMullin number. In the case of the bending experiment, this reduction could already have happened when the composite was manufactured and might therefore be negated by the change in the MacMullin number. Another explanation is based on the mechanics. Theoretically, shear forces are not present between the inner force entry point and therefore the area of the supercapacitor. In reality, shear forces can occur and therefore the supercapacitors might be compressed, leading to an uneven distance between its electrodes. Both hypotheses will be investigated in future studies.

Beside R_S_, the resistors R_1_, R_2_, and R_3_ increase with mechanical load in both the 4PB experiment and the pure pressure setup. These resistors coupled with CPE elements in the ECM describe a charge transfer reaction. A rising resistance indicates a hampered charge transfer. It is influenced by the exchange current density, which depends on the concentration of oxidized and reduced species. In a pore network, the diffusion is influenced by the porosity [34], the tortuosity [35], and the constrictivity [36]. The latter describes the influence on the motion of ions through small pores. If this is influenced by the mechanical load, the concentration could change and thus also the charge transfer. This explanation is only valid for small-sized pores such as the ones described by R_3_. A change in porosity and tortuosity is also a possibility for pores of all sizes. With the data available in this work, the influence factors cannot be separated. However, a change in porosity as well as tortuosity is likely, as both are affected when the volume is changed.

## 5. Conclusions

In this work, we describe the use of structure integrated supercapacitors as mechanical strain sensors for condition monitoring. First, we analyze supercapacitors in a pure pressure setup by carrying out in situ electrochemical impedance spectroscopy at ten different load states. This spectroscopy is analyzed by fitting an electrochemical model. To evaluate that model, we carried out mercury porosimetry measurements, scanning electron microscopy and a frequency analysis. The results suggested that the pores of the supercapacitor’s electrode change their geometry through mechanical load, while, at the same time, the porosity, tortuosity and constrictivity also changes. To show supercapacitors can work as a strain sensor in composite materials, structure integrated ones are manufactured.

Therefore, supercapacitors are integrated in glass fiber reinforced composites, which are investigated in four-point bending. The supercapacitors are integrated in the tension and pressure side to determine the influence of different load scenarios. The electrochemical process is investigated with in situ electrochemical impedance spectroscopy. Finally, it is possible to detect mechanical strain down to 0.2% by the integrated supercapacitors. Since the effects are reversible, the system can be used as a multifunctional sensor system that is able to store electric energy and act as a strain sensor. Through novel design approaches it is possible to reduce system weight and the volume of future transport systems by using multifunctional materials that can deliver electric energy and provide a load carrying structure with intrinsic load monitoring information. The mechanisms described in this work can be the basis for such a load monitoring material.

To bring that system into use, further studies are needed. For example, the influence of temperature should be investigated to extend the described model, making it more resistant against temperature changes.

## 6. Verification and Limitations of the Measurement

Due to the complexity of the experiment, six samples with two supercapacitors each were investigated. All of them were measured by EIS under mechanical load in order to determine their behavior under bending load.

To ensure high quality measurements and fitting results, the measurements were conducted with respect to [31], while the fitting was carried out with the help of [37]. This means that the placement of cables was optimized to reduce self-induction. Furthermore, the machine was calibrated, and the measurement was performed within the accuracy plot in order to reach an error smaller than one percent [30]. Electrochemical impedance spectroscopy was carried out while the sample was placed once inside and once outside the mechanical test setup to determine the influence of the setup, which can be neglected.

The residuals in the fitting results are random and the errors of the equivalent elements are small. Additionally, the model is based on a physical background [37].

A change in electric resistance between the current collector and the electrode during the experiment and, therefore, a change in impedance, is less likely, as the impedance spectra before and after the experiment (both in load free stages) still overlap. Therefore, an ageing effect can be excluded.

One of the six samples deviated strongly from the others and was therefore excluded from the analysis.

## 7. Patents

[DE] Strukturmonitoring mittels Impedanzspektroskopie an einem strukturintegrierten Energiespeicher; DE102020104584B3.

## Data Availability

Not applicable.

## Supplementary Materials

The following supporting information can be downloaded at: https://www.mdpi.com/article/10.3390/s22186932/s1, Figure S1: Test setup to characterize the electrodes under pressure. The electrodes are placed inside an alu-minum container filled with electrolyte. Pressure is transferred with an aluminum stamp con-nected to a test machine. Force is measured with a load cell. EIS in situ measurement is carried out with a Potentiostat connected via crocodile clamps to the specimen. Figure S2: Cyclic Voltammetry of the sample. Scan rate is set to 20 mV/s. The capacity calculated in the negative discharge area (grey field) is 115 mF.
