# Supplementary material for: Structure-Integrated Thin-Film Supercapacitor as a Sensor
Source: Sensors (Basel). 2022 Sep 13;22(18):6932. doi: 10.3390/s22186932 (PMC9504183; doi:10.3390/s22186932)
Supplement: Supplementary file 1 [file sensors-22-06932-s001.zip › sensors-1873109-supplementary.pdf]

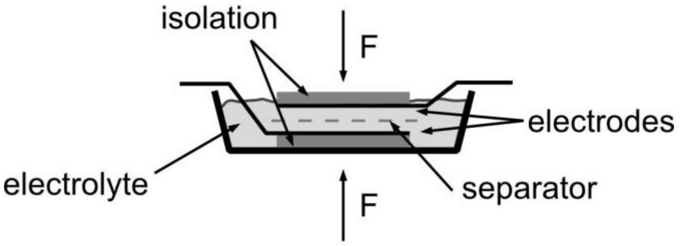

Figure S1: Test setup to characterise the electrodes under pressure. The electrodes are placed inside an aluminium container filled with electrolyte. Pressure is transferred with an aluminium stamp connected to a test machine. Force is measured with a load cell. EIS in situ measurement is done with a Potentiostat connected via crocodile clamps to the specimen.

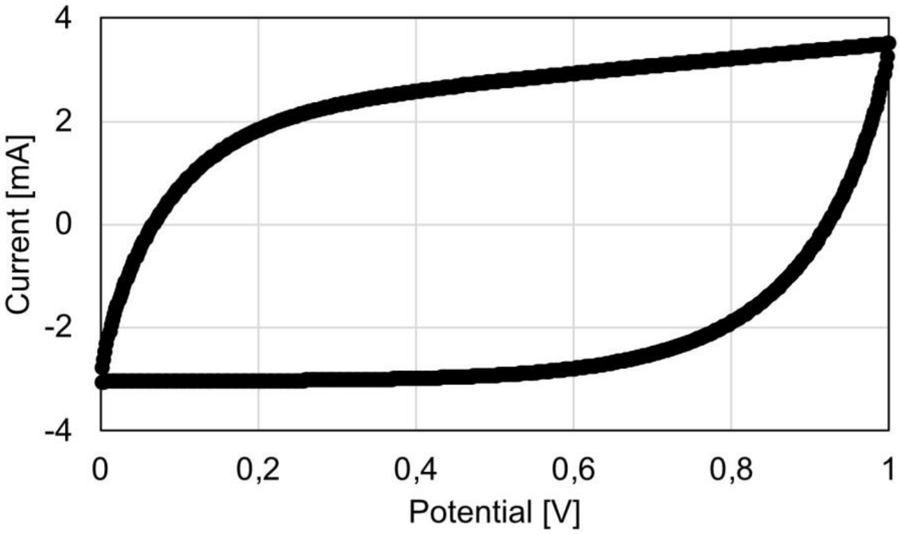

Figure S2: Cyclic Voltammetry of the sample. Scan rate is set to 20 mV/s. The capacity calculated in the negative discharge area (grey field) is 115 mF.
